# Parvovirus B19 Associated Hepatitis

**DOI:** 10.1155/2013/472027

**Published:** 2013-10-22

**Authors:** Chhagan Bihari, Archana Rastogi, Priyanka Saxena, Devraj Rangegowda, Ashok Chowdhury, Nalini Gupta, Shiv Kumar Sarin

**Affiliations:** ^1^Department of Pathology, Institute of Liver and Biliary Sciences, D-1, Vasant Kunj, New Delhi 110070, India; ^2^Department of Hematology, Institute of Liver and Biliary Sciences, D-1, Vasant Kunj, New Delhi 110070, India; ^3^Department of Hepatology, Institute of Liver and Biliary Sciences, D-1, Vasant Kunj, New Delhi 110070, India

## Abstract

Parvovirus B19 infection can present with myriads of clinical diseases and syndromes; liver manifestations and hepatitis are examples of them. Parvovirus B19 hepatitis associated aplastic anemia and its coinfection with other hepatotropic viruses are relatively underrecognized, and there is sufficient evidence in the literature suggesting that B19 infections can cause a spectrum of liver diseases from elevation of transaminases to acute hepatitis to fulminant liver failure and even chronic hepatitis. It can also cause fatal macrophage activation syndrome and fibrosing cholestatic hepatitis. Parvovirus B19 is an erythrovirus that can only be replicate in pronormoblasts and hepatocytes, and other cells which have globosides and glycosphingolipids in their membrane can also be affected by direct virus injury due to nonstructural protein 1 persistence and indirectly by immune mediated injury. The virus infection is suspected in bone marrow aspiration in cases with sudden drop of hemoglobin and onset of transient aplastic anemia in immunosuppressed or immunocompetent patients and is confirmed either by IgM and IgG positive serology, PCR analysis, and in situ hybridization in biopsy specimens or by application of both. There is no specific treatment for parvovirus B19 related liver diseases, but triple therapy regimen may be effective consisting of immunoglobulin, dehydrohydrocortisone, and cyclosporine.

## 1. Background

Parvoviridae family includes many pathogenic animal viruses including adeno-associated viruses which appear to infect humans without causing clinical manifestations. Most parvoviruses depend upon the help from host cells or other viruses to replicate, whereas only few (autonomous) parvoviruses propagate in actively dividing cells. Parvovirus B19 (B19) is the type member of the erythrovirus genus which propagates primarily in erythroid progenitor cells [1]. 

B19 can infect erythroid precursors, hepatocytes, and other cells that possess globosides and glycosphingolipids in their cell membrane, but it can only replicate in the erythroid precursors and few other cells including fetal liver, isolated stem and bone marrow cells, and megakaryocytic leukemia cell lines maintained with erythropoietin [2, 3]. 

Infection of parvovirus B19 is globally prevalent with infection being very common among children. The virus spreads primarily through respiratory droplets, and secondary infection is by household contacts. It can also be transmitted as nosocomial infections and by blood products. B19 is resistant to heat inactivation and organic detergent, because of their stable genomic structure and absence of lipid envelope [1]. 

B19 is an etiologic agent of erythema infectiosum (fifth disease), fever/rash illness of childhood, whereas, in adults, the commonest manifestation is clinically significant arthropathy [1–3]. Both of these clinical diseases are thought to be due to immune complex deposition in skin and in the joints, respectively [3–5]. Systemic manifestation of B19 infection includes multisystem involvement and viral hemophagocytic syndrome [2]. Ever expanding spectrum of clinical disease has been attributed to human B19 infection with adult seroprevalence rate of around 50% [1]. 

The clinical diseases caused by B19 are categorized into two broad groups—common and uncommon. Common clinical diseases are the ones listed in Table 1 [1], and the uncommon clinical manifestations associated with B19 are enlisted in Table 2 [6–14]. 

Acute hepatitis and fulminant liver failure may be caused by B19; however, this incidence is very rare [2], with only a few cases reported in the literature with clinical manifestation of hepatitis as a result of B19 infection. We undertook this facet of B19 infection for the discussion and reviewed various liver related conditions along with diagnosis, pathogenesis, and treatment of B19 induced hepatitis.

## 2. Parvovirus B19 and Hepatitis

**Figure 1:**
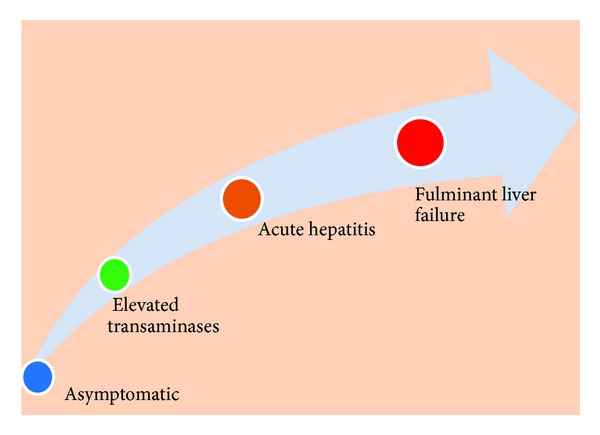
Diagrammatic representation of spectrum of liver diseases associated with parvovirus B19 infection according to the severity.

Liver diseases caused by B19 infection range from elevation of transaminases to acute hepatitis to fulminant liver failure and even chronic hepatitis (Figure 1). According to a study by Mihály et al., parvovirus B 19 related hepatitis may occur in 4.1% of patients infected by this virus [15]. Around 50 cases of B19 related hepatitis have been reported in the literature till date which is summarized in Table 3 [16–53]. Spectrum of liver diseases has been reported in all age groups from neonates to elderly. 

### 2.1. Parvovirus B19 Acute Hepatitis and Fulminant Hepatic Failure

**Figure 2:**
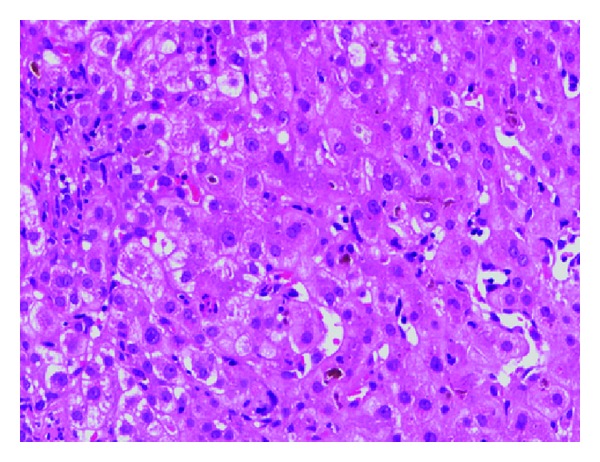
Liver biopsy showing features of acute cholestatic hepatitis (H&E, 200x). The patient was a case of thalassemia trait, and parvovirus B19 IgM serology was positive.

Presentation as acute hepatitis or fulminant liver failure has been mostly reported in the paediatric age groups; however, the same has also been reported in adults. In adults, parvovirus B19 hepatitis course is found to be less severe than in children [16–22] and can be manifested in immunocompetent or immunodeficient patients with or without underlying hemolytic abnormalities [25]. Most of the time, B19 acute hepatitis shows complete and spontaneous remission, particularly in adults [25]. Fulminant hepatic failure induced as a result of acute B19 infection remains a rare clinical entity. And these may be underreported also due to infrequent testing and lack of awareness [25, 26]. Liver biopsy in affected patients displays cellular and canalicular cholestasis, apoptosis (Figure 2), and variable amounts of necrosis depending upon immune status of the host and the severity of liver involvement [19].

### 2.2. Parvovirus B19 and Chronic Hepatitis

B19 can also cause chronic hepatitis. In a case by Mogensen et al., chronic hepatitis due to B19 was reported in a patient with lymphopenia [23]. Pongratz et al. found that the persistence of B19 and occurrence of chronic hepatitis directly correlate with the extent of liver involvement [27]. Wang et al. described B19 persistence in the chronic hepatitis B (CHBV) and chronic hepatitis C (CHCV) infected patients and concluded that the persistence of B19 virus infection does not cause any significant worsening of liver functions in the HBV and HCV affected patients [24]. A study by Toan et al. on 463 hepatitis B positive Vietnamese patients showed that 99/463 patients (21.4%) were positive for B19 DNA which was significantly higher than those of healthy controls. They also concluded that in HBV/B19 coinfection the probability of progression to more severe hepatitis is significantly higher [54]. 

The association of B19 with chronic hepatitis B and C has also been described by Hsu et al. They found that B19 serology for IgM and IgG was positive in 35.2% and 85%. 2% of the cases of chronic hepatitis B with B19 DNA were detected in 37% of the cases of chronic hepatitis B. In cases of chronic hepatitis C, IgM and IgG antibodies for B19 were positive in 15.7% and 70.6%, and B19 DNA was detected in 23.5% of HCV cases. Distinctive subtypes of B19 were detected in chronic hepatitis B and C, TW-3 in chronic hepatitis B and TW-9 in cases of chronic hepatitis C infection. Liver dysfunction was not associated with B19 coexistence in the chronic hepatitis cases. The study also revealed that a significant proportion of coinfection occurs in chronic hepatitis cases with B19 infection [55]. It is important to note that although liver functions are not much affected by the coinfection of B19 [24, 55], large cohort studies are required to explore the pathological course of B19 in association with chronic hepatitis and their clinical outcomes. 

### 2.3. Parvovirus B19 and Fibrosing Cholestatic Hepatitis

There was a single case report of fibrosing cholestatic hepatitis (FCH) due to B19 infection in a patient with renal allograft for IgA nephropathy. During the postoperative period, the patient developed features of acute liver failure. All viral markers were negative except for HBV and B19 DNA. The patient was given lamivudine therapy; however, his condition got deteriorated, and the patient subsequently died. The postmortem liver tissue revealed FCH. On immunohistochemical examination, the biopsy was negative for HBsAg and HBcAg, while the PCR showed strong positivity in liver tissue for B19 infection, and it was considered that B19 was the cause of FCH [40]. 

### 2.4. Parvovirus B19 Coinfection with Other Hepatotropic Viruses

 B19 coinfection with other hepatotropic viruses can lead to severe acute fulminant hepatic failure (FHF) with severe outcome as compared to isolated B19 or other hepatotropic virus associated FHF. Dwivedi et al. in their study of 48 patients with FHF, divided them into three groups as those associated with (i) B19 infection alone, (ii) one or more other hepatotropic viral infection in the absence of B19 infection, and (iii) B19 coinfection with other hepatotropic viruses. They found that FHF caused by B19 and coinfection with other hepatitis viruses had severe jaundice, high bilirubin, high alanine aminotransferase or aspartate aminotransferase activity, and unfavorable outcome resulting in death of most of these patients, compared with those with isolated B19 or other hepatitis viruses infection [56]. It was hypothesized that B19 possibly may cause injury to hepatocytes independently or by producing synergistic effect when present along with other hepatitis viruses. 

### 2.5. Parvovirus B19 and Hepatitis Associated Aplastic Anemia (HAAA)

Hepatitis associated aplastic anemia (HAAA) is a distinct variant of acquired aplastic anemia (AA), in which an acute attack of hepatitis culminates in marrow failure and pancytopenia [57]. Several hepatitis viruses such as hepatitis A, B, C, E, and G have been anticipated to be associated with this set of symptoms. Besides the hepatitis viruses, other viruses have also been implicated as causative agent of AA which include B19, *Cytomegalovirus*, Epstein Barr virus, echovirus 3, GB virus-C, transfusion transmitted virus (TTV), SEN virus, and non-A-E hepatitis virus (unknown viruses). B19, an underrecognized hepatotropic virus, is documented as an offending agent of acute hepatitis, FHF, and HAAA in immunocompromised patients [58]. There have been reports of B19 related HAAA in post-liver-transplant immunocompromised patients [48, 52]. The myelotoxic effect in B19 or other viral infections is thought to be due to increased circulating cytotoxic CD8^+^ T cells and IFN-*γ* secretion by these cells. Similarly, high circulating CD8^+^ T cells cause the altered and defective monocyte and macrophage differentiation, decreased level of circulating IL-1, and increased secretion of TNF-*α*, IFN-*γ*, and IL-2 receptors which causes onlooker damage of hepatocytes and subsequently occurrence of acute hepatitis [59, 60].

### 2.6. Parvovirus B19 Hepatitis in Underlying Hematological Diseases

In patients with hemolytic anemia, B19 infection can cause an abrupt cessation of red cell production which is exacerbated in case of acute infection or in compensated states and provokes severe anemia. Anemic crisis in hereditary spherocytosis and in sickle cell disease has long been recognized. The bone marrow in patients with transient aplastic crisis is characterized by an absence of maturing erythroid precursors and presence of giant pronormoblasts. Thrombocytopenia and pancytopenia have also been reported in patients with acute B19 infection [2]. Transient aplastic crisis (TAC) is a self-limiting condition, and normal individuals usually recover as the neutralizing antibodies are produced, while in immunocompromised patients, neutralizing antibody cannot be produced, and this can lead to persistent pure red aplasia [1]. Zaki et al. found that the incidence of B19 infection is significantly higher among children with hematological disorders, including hemolytic anemias, lymphomas, and leukemias on chemotherapy [61]. B19 has a tropism for the immature proliferating pronormoblasts and is essentially an erythrovirus. Globoside, a neutral glycolipid that acts as a cellular receptor and nonstructural protein of parvovirus, is responsible for the apoptotic death of erythroid progenitors, and some other cells such as megakaryocytes may be lysed by restricted expression of viral proteins in the absence of viral propagation [62]. Thus, the high propensity of B19 in hematological disease at times can also cause acute hepatitis [63].

### 2.7. Parvovirus B19 Hepatitis and Hemophagocytosis Lymphohistiocytosis (HLH)

Hemophagocytic lymphohistiocytosis (HLH) is a hyperinflammatory condition clinically characterized by fever, splenomegaly, jaundice, and phagocytosis of erythrocytes, leukocytes, platelets, and their precursors by macrophages in bone marrow and other tissues [64]. Among viruses, Epstein-Barr virus, *Cytomegalovirus*, human herpesvirus 6, and parvovirus B19 have been implicated to cause virus associated HLH (VAHLH) [65]. B19 related HLH is not so uncommon and can occur both in immunocompetent and immunocompromised hosts [66]. The hallmark of HLH pathogenesis is T cell activation leading to stimulation of macrophages which thereby initiates hemophagocytosis. Thus, CD8^+^ and CD4^+^ T cells activation triggers marked cytokine production of TNF-*α*, a key factor in histiocytic activation [67]. As described above, activated T cells and released cytokines may also lead to hepatocyte damage and ultimately hepatitis.

## 3. Diagnosis

### 3.1. Hematological Tests

**Figure 3:**
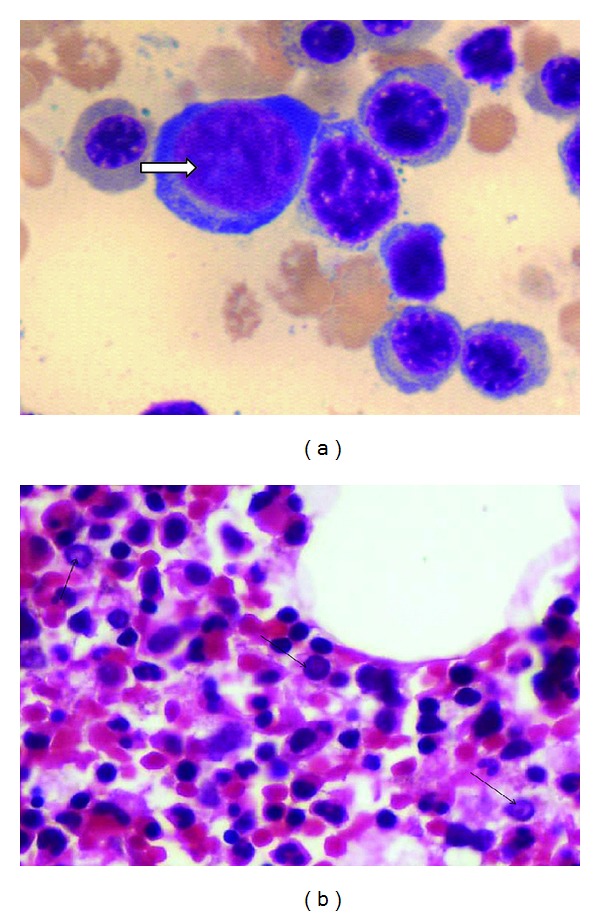
(a) Parvovirus B19 inclusion in Pronormoblast in bone marrow aspirate (Geimsa, 1000x), in same case as described above. (b) Clearing of nuclei in pronormoblasts, due to Parvovirus B19 inclusion (HE, 400x); an adult case of hereditary spherocytosis with acute hepatitis and sudden drop of haemoglobin, Parvovirus IgM serology positive.

In patients with evidence of clinically significant anemia or transient aplastic crisis (TAC), a complete blood count with reticulocyte count aids in suspecting B19 infection with the following possible scenarios: (1) patients infected with parvovirus B19 will have a low reticulocyte count (0-1%) and (2) in an aplastic crisis, hemoglobin levels will drop below the patient's baseline by at least 2 g/dL [68]. Bone marrow examination in these patients reveals absence of maturing red cell with prominence of pronormoblast having cytoplasmic blebs and intranuclear inclusions (Figure 3(a)). Bone marrow biopsy examination shows nuclear clearing due to viral inclusions present in them [69] (Figure 3(b)). 

### 3.2. Serology

Parvovirus serology (anti-parvovirus B19 immunoglobulin M (IgM) and immunoglobulin G (IgG) antibodies) can be determined using enzyme-linked immunoassay (ELISA), radioimmunoassay, or immunofluorescence. Results of IgM testing are maybe difficult to interpret; however, reliable results can be obtained by using automated instruments dedicated for serological testing. Generally, IgM antibodies are detectable 3 days after infection, and IgG antibodies can be detected after 2 weeks at the time of recovery of hematopoiesis. IgM antibodies once formed remain detectable for months, while IgG can be detected for lifetime. In immunodeficient patients, inability to clear the virus leads to chronic B19 infection and leads to pure red cell aplasia (PRAC). In contrast to TAC, PRAC is characterized by very low or absent antibody levels, and they are diagnosed best by polymerase chain reaction (PCR) [70]. Pregnant women exposed to parvovirus B19 should get IgG and IgM serology done as soon as possible, as infection risk for fetus remains due [71]. 

### 3.3. Polymerase Chain Reaction (PCR)

PCR testing for parvovirus B19 is routinely available with high sensitivity level. Low levels of B19 DNA can be detected for more than 4 months in serum after acute infection and for years in other tissues. PCR can also be used to diagnose chronic infection by detecting viral DNA present in the blood or other tissues/fluids. However, the interpretation pertaining to pregnant women is uncertain [72]. 

### 3.4. Immunohistochemistry

Parvovirus B19 monoclonal antibody R92F6 against VP1/VP2 capsid protein antigen can also be detected in liver tissue and in bone marrow biopsy by immunohistochemistry [39].

### 3.5. Other Potential Diagnostic Methods

Enzyme-linked immunosorbent spot assay (ELISPOT) is a method to measure the qualitative and quantitative immune response in humans and animals. This method identifies and enumerates cytokine-producing cells at the single cell level. By having appropriate conditions, the ELISPOT assay allows visualization of the secretory product of individual activated or responding cells [27]. Each spot that develops in the assay represents a single reactive cell. This ELISPOT technique can be used to detect CD4^+^ T cells specific for B19 viral proteins in cases of persistent infection [27].

The diagnosis of acute or chronic infection should be made on the basis of standard DNA hybridization or quantitative (real-time) PCR in combination with serologic assays for B19-specific IgG, IgM, or both [73]. 

## 4. Pathogenesis

The mechanism by which parvovirus B19 infection may result in hepatic injury is exactly not clear. Hepatic cell damage related to direct viral invasion is one possibility. Alternatively, injury may result as an indirect consequence of the immune response directed against the virus.

### 4.1. Direct Cytopathic Effect

**Figure 4:**
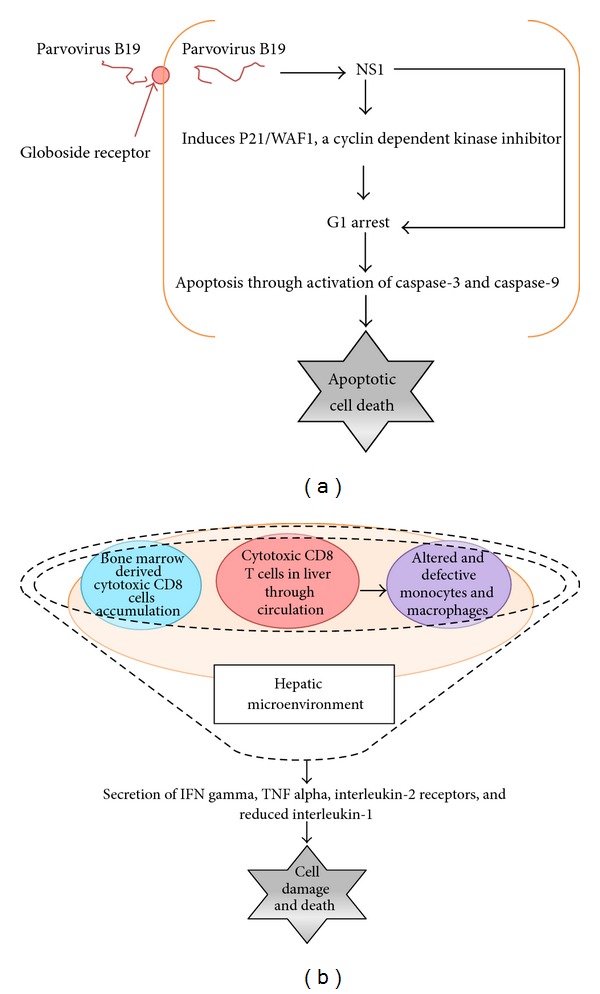
Schematic presentation of direct and indirect hepatocellular injury in parvovirus B19 infection.

B19 is a single-stranded DNA virus and has a genome length of 5.4 kb with hairpin structures at each extremity. Two major open reading frames (ORFs) extend through the entire genome of virus. A nonstructural protein (NS1) is found on the N-terminal region of the genome, and its molecular weight is 70 to 77 kDa. NS1 is thought to be essential for viral DNA replication and also for the regulation of viral promoters. NS1 contains a consensus sequence for ATP- or GTP-binding, which is associated with ATPase and DNA helicase activities. NS1 is also known to be cytotoxic for erythroid cells and is possibly related to the pathogenesis of B19 virus infection [74]. B19 can replicate only in the erythroid precursors and few other cells including fetal liver, isolated stem and bone marrow cells, and megakaryocytic leukemia cell lines maintained with erythropoietin. Hepatocytes express globoside and glycosphingolipids, the putative receptors for B19 virus. B19 virus enters hepatocytes through globoside and establishes a restricted infection with the production of NS1 without the production of viral progeny [75]. NS1 expression plays a critical role in G1 arrest induced by B19 virus. Furthermore, NS1 expression also significantly increases p21/WAF1 expression, a cyclin dependent kinase inhibitor that induces G1 arrest. Ultimately, the G1 arrested hepatocytes undergo apoptosis by activation of caspase-3 and caspase-9 [74, 76]. Diagrammatic summary is depicted in Figure 4(a).

### 4.2. Indirect Immunological Effect

The hepatotoxic effect caused by parvovirus B19 infection is thought to be due to increased circulating CD8^+^ cytotoxic T cells and IFN-*γ* and TNF-*α* secretion by these cells [58]. Similarly, high circulating CD8^+^ T cells cause the altered and defective monocyte and macrophage differentiation, decreased level of circulating IL-1, and increased secretion of TNF-*α*, IFN-*γ*, and IL-2 receptors which causes damage of hepatocytes and subsequently leading to acute hepatitis [58–60]. Diagrammatic summary is depicted in Figure 4(b).

## 5. Treatment

There are no specific treatment guidelines for infection caused by B19 virus, and most of the symptoms and elevation of liver enzymes presented during infection stage resolve without any treatment. In case of acute and fulminant hepatitis, combination therapy consisting of an intravenous infusion of immunoglobulin and dehydrohydrocortisone and subcutaneous injections of granulocyte colony-stimulating factor for three months has been tried [17]. For HAAA and HLH, an immunosuppressive therapy comprising antithymocyte Globulin (ATG), cyclosporine, and steroids has proven to be effective [58]. Aplastic crises and PRAC are transiently responsive to erythropoietin, growth factors, granulocyte colony-stimulating factor, granulocyte macrophage colony stimulating factor, interleukin-3, and androgens [77]. Nonresponsive HAAA should be treated by allogenic bone marrow (BM) transplantation from HLA matched siblings [78]. 

## 6. Conclusion

There are sufficient lines of evidence in the literature that state that Parvovirus B19 infection can be associated with the development of acute hepatitis, FHF, HAAA, hepatitis with HLH, chronic hepatitis, and rarely FCH. There is a significant rate of coexistence of B19 with chronic hepatitis B and C as suggested by the literature. This area needs to be further explored and validated through large cohort studies. Infection with parvovirus B19 should be considered in the differential diagnosis in both immunocompromised and immunocompetent patients presenting with acute hepatitis of unknown etiology particularly in cases of underlying hemolytic diseases and immunodeficient host with aplastic anemia. The parvovirus B19 infection can be detected by positive IgM serology and by PCR in infected tissues. Parvovirus B19 can cause hepatitis due to direct cytopathic and indirect immunological injury through CD8^+^ cytotoxic T cells. A combination of an intravenous infusion of immunoglobulin, dehydrohydrocortisone and cyclosporine and subcutaneous injections of granulocyte colony-stimulating factor for three months has proved to be an effective therapy for parvovirus B19 hepatitis and HAAA. 

## Figures and Tables

**Table 1:** Common clinical manifestations of parvovirus B19.

Diseases	Group of patients
Fifth disease	Children
Arthropathy	Adults
Transient aplastic crisis	Patients with increased erythroid proliferation (underlying hemolytic disease)
Persistent anemia	Patients with immunocompromised or immunodeficient status
Hydrops fetalis	Fetus

**Table 2:** Uncommon clinical diseases associated with parvovirus B19.

Clinical disease	References
Hepatitis	[6]
Myocarditis	[7]
Necrotizing vasculitis	[8]
Kawasaki's disease	[9]
Henoch-Schönlein purpura	[10]
Giant-cell arteritis	[11]
Gloves-and-socks syndrome	[12]
Chronic fatigue syndrome	[13]
Meningitis	[14]
Encephalitis	[14]
Ophthalmitis	[14]

**Table 3:** List of reported cases of parvovirus B19 hepatitis.

Author	Cases	Associated condition	References
Martínez González et al. (2012)	One (acute hepatitis)		[16]
Sun and Zhang (2012)	One (FHF)	Aplastic anemia	[17]
Larsen (2011)	One (acute hepatitis)		[18]
Hatakka et al. (2011)	One (acute hepatitis)		[19]
Yang et al. (2012)	One (acute hepatitis)	DLBCL	[20]
Sun et al. (2011)	Two (acute hepatiti)		[21]
Al Nahdi et al. (2010)	One (recurrent acute hepatitis)		[22]
Mogensen et al. (2010)	One (chronic hepatitis)	Lymphopenia	[23]
Wang et al. (2009)	One (chronic hepatitis)		[24]
Krygier et al. (2009)	One (acute hepatitis)		[25]
Kim et al. (2009)	One (acute hepatitis)		[26]
Pongratz et al. (2009)	One (acute hepatitis)		[27]
Cao et al. (2009)	One (fulminant hepatic failure)		[28]
Kishore and Sen (2009)	One (fulminant hepatic failure)	Coexistent A and E	[29]
Al-Abdwani et al. (2008)	One (acute hepatitis)	Aplastic anemia	[30]
Giørtz-Carlsen et al. (2007)	One (acute hepatitis)		[31]
Özçay et al. (2006)	One (fulminant hepatic failure)	Pure red cell aplasia	[32]
Aydin et al. (2006)	One (acute hepatitis)		[33]
Toshihiro et al. (2003)	One (acute hepatitis)		[34]
Chehal et al. (2002)	One (acute hepatitis)		[35]
Dame et al. (2002)	One (acute hepatitis)	Aplastic anemia	[36]
Díaz and Collazos (2000)	One (acute hepatitis)		[37]
Lee et al. (2000)	One (acute hepatitis)	Post-renal-transplant immunosuppression	[38]
Pinho et al. (2001)	One (acute hepatitis)		[39]
Shan et al. (2001)	One (FCH)	Post-renal-transplant immunosuppression	[40]
Alliot et al. (2001)	One (acute hepatitis)	HIV	[41]
Karetnyi et al. (1999)	One (fulminant hepatic failure)		[42]
Drago et al. (1999)	One (acute hepatitis)		[43]
Sokal et al. (1998)	One (fulminant hepatic failure)		[44]
Hillingsø et al. (1998)	One (acute hepatitis)		[45]
Hillingsø et al. (1998)	One (acute hepatitis)		[46]
Longo et al. (1998)	One (acute hepatitis)	Still's disease	[47]
Pardi et al. (1998)	Two (acute hepatitis)	Post-Liver-transplant aplastic anemia	[48]
Weinberg et al. (1996)	One (acute hepatitis)		[49]
Naides et al. (1996)	One (acute hepatitis)		[50]
Yoto et al. (1996)	One (acute hepatitis)		[51]
Langnas et al. (1995)	Six (2 acute hepatitis, 4 fulminant hepatitis)	Aplastic anemia	[52]
Pouchot et al. (1993)	One (acute hepatitis)		[53]

## References

[1] Young NS, Brown KE (2004). Mechanisms of disease: parvovirus B19. *The New England Journal of Medicine*.

[2] Brown KE, Young NS (1997). The simian parvoviruses. *Reviews in Medical Virology*.

[3] Moore TL (2000). Parvovirus-associated arthritis. *Current Opinion in Rheumatology*.

[4] Chorba T, Coccia P, Holman RC (1986). The role of parvovirus B19 in aplastic crisis and erythema infectiosum (fifth disease). *Journal of Infectious Diseases*.

[5] He Z, Zhuang H, Wang X (2003). Retrospective analysis of non-A-E hepatitis: possible role of hepatitis B and C virus infection. *Journal of Medical Virology*.

[6] Arista S, De Grazia S, Di Marco V, Di Stefano R, Craxì A (2003). Parvovirus B19 and “cryptogenic” chronic hepatitis. *Journal of Hepatology*.

[7] Beghetti M, Gervaix A, Haenggeli CA, Berner M, Rimensberger PC (2000). Myocarditis associated with parvovirus B19 infection in two siblings with merosin-deficient congenital muscular dystrophy. *European Journal of Pediatrics*.

[8] Finkel TH, Török TJ, Ferguson PJ (1994). Chronic parvovirus B19 infection and systemic necrotising vasculitis: opportunistic infection or aetiological agent?. *The Lancet*.

[9] Nigro G, Zerbini M, Krzysztofiak A (1994). Active or recent parvovirus B19 infection in children with Kawasaki disease. *The Lancet*.

[10] Ferguson PJ, Saulsbury FT, Dowell SF, Török TJ, Erdman DD, Anderson LJ (1996). Prevalence of human parvovirus B19 infection in children with Henoch-Schönlein purpura. *Arthritis and Rheumatism*.

[11] Gabriel SE, Espy M, Erdman DD, Bjornsson J, Smith TF, Hunder GG (1999). The role of parvovirus B19 in the pathogenesis of giant cell arteritis: a preliminary evaluation. *Arthritis and Rheumatism*.

[12] Smith SB, Libow LF, Elston DM, Bernert RA, Warschaw KE (2002). Gloves and socks syndrome: early and late histopathologic features. *Journal of the American Academy of Dermatology*.

[13] Kim Jacobson S, Daly JS, Thorne GM, McIntosh K (1997). Chronic parvovirus B19 infection resulting in chronic fatigue syndrome: case history and review. *Clinical Infectious Diseases*.

[14] Kerr JR, Barah F, Chiswick ML (2002). Evidence for the role of demyelination, HLA-DR alleles, and cytokines in the pathogenesis of parvovirus B19 meningoencephalitis and its sequelae. *Journal of Neurology Neurosurgery and Psychiatry*.

[15] Mihály I, Trethon A, Arányi Z (2012). Observations on human parvovirus B19 infection diagnosed in 2011. *Orvosi Hetilap*.

[16] Martínez González J, Senosiain Lalastra C, Mesonero Gismero F, Moreira Vicente V (2012). An exceptional cause of acute hepatitis in an adult: parvovirus B19. *Journal of Gastroenterology and Hepatology*.

[17] Sun L, Zhang J-C (2012). Acute fulminant hepatitis with bone marrow failure in an adult due to parvovirus B19 infection. *Hepatology*.

[18] Larsen L (2011). Parvovirus B19-akut hepatitis hos immunkompetent patient. *Ugeskrift for Laeger*.

[19] Hatakka A, Klein J, He R, Piper J, Tam E, Walkty A (2011). Acute hepatitis as a manifestation of parvovirus B19 infection. *Journal of Clinical Microbiology*.

[20] Yang S-H, Lin L-W, Fang Y-J, Cheng A-L, Kuo S-H (2012). Parvovirus B19 infection-related acute hepatitis after rituximab-containing regimen for treatment of diffuse large B-cell lymphoma. *Annals of Hematology*.

[21] Sun L, Zhang J-C, Jia Z-S (2011). Association of parvovirus B19 infection with acute icteric hepatitis in adults. *Scandinavian Journal of Infectious Diseases*.

[22] Al Nahdi N, Wiesinger H, Sutherland H, Yoshida EM (2010). Recurrent idiopathic acute hepatitis-associated aplastic anemia/pancytopenia fourteen years after initial episode. *Annals of Hepatology*.

[23] Mogensen TH, Jensen JMB, Hamilton-Dutoit S, Larsen CS (2010). Chronic hepatitis caused by persistent parvovirus B19 infection. *BMC Infectious Diseases*.

[24] Wang C, Heim A, Schlaphoff V (2009). Intrahepatic long-term persistence of parvovirus B19 and its role in chronic viral hepatitis. *Journal of Medical Virology*.

[25] Krygier DS, Steinbrecher UP, Petric M (2009). Parvovirus B19 induced hepatic failure in an adult requiring liver transplantation. *World Journal of Gastroenterology*.

[26] Kim BJ, Yoo KH, Li K, Kim MN (2009). Parvovirus B19 infection associated with acute hepatitis in infant. *Pediatric Infectious Disease Journal*.

[27] Pongratz G, Lindner J, Modrow S, Schimanski S, Schölmerich J, Fleck M (2009). Persistent parvovirus B19 infection detected by specific CD4+ T-cell responses in a patient with hepatitis and polyarthritis. *Journal of Internal Medicine*.

[28] Cao Y-H, Zhang G-Y, Zhang G-C (2009). Successful treatment with high-dose intravenous immunoglobulin for parvovirus B19 infection associated with acute fulminant hepatitis in a chinese child. *Clinical Pediatrics*.

[29] Kishore J, Sen M (2009). Parvovirus B19-induced thrombocytopenia and anemia in a child with fatal fulminant hepatic failure coinfected with hepatitis A and E viruses. *Journal of Tropical Pediatrics*.

[30] Al-Abdwani RM, Khamis FA, Balkhair A, Sacharia M, Wali YA (2008). A child with human parvovirus B19 infection induced aplastic anemia and acute hepatitis: effectiveness of immunosuppressive therapy. *Pediatric Hematology and Oncology*.

[31] Giørtz-Carlsen B, Rittig S, Thelle T (2007). Neurological symptoms and acute hepatitis associated with parvovirus B19. *Ugeskrift for Laeger*.

[32] Özçay F, Bikmaz YE, Canan O, Özbek N (2006). Hepatitis A and parvovirus B19 infections in an infant with fulminant hepatic failure. *Turkish Journal of Gastroenterology*.

[33] Aydin M, Bulut Y, Poyrazoglu G, Turgut M, Seyrek A (2006). Detection of human parvovirus B19 in children with acute hepatitis. *Annals of Tropical Paediatrics*.

[34] Toshihiro M, Takikawa Y, Fukuda Y, Sato S-I, Endou R, Suzuki K (2003). A case of acute hepatitis associated with Parvovirus B19. *Japanese Journal of Gastroenterology*.

[35] Chehal A, Sharara AI, Haidar HA, Haidar J, Bazarbachi A (2002). Acute viral hepatitis A and parvovirus B19 infections complicated by pure red cell aplasia and autoimmune hemolytic anemia. *Journal of Hepatology*.

[36] Dame C, Hasan C, Bode U, Eis-Hübinger AM (2002). Acute liver disease and aplastic anemia associated with the persistence of B19 DNA in liver and bone marrow. *Pediatric Pathology and Molecular Medicine*.

[37] Díaz F, Collazos J (2000). Hepatic dysfunction due to parvovirus B19 infection. *Journal of Infection and Chemotherapy*.

[38] Lee PC, Hung CJ, Lei HY, Chang TT, Wang JR, Jan MS (2000). Parvovirus B19-related hepatitis in an immunosuppressed kidney transplant. *Nephrology Dialysis Transplantation*.

[39] Pinho JRR, Alves VAF, Vieira AF (2001). Detection of human parvovirus B19 in a patient with hepatitis. *Brazilian Journal of Medical and Biological Research*.

[40] Shan Y-S, Lee P-C, Wang J-R, Tsai H-P, Sung C-M, Jin Y-T (2001). Fibrosing cholestatic hepatitis possibly related to persistent parvovirus B19 infection in a renal transplant recipient. *Nephrology Dialysis Transplantation*.

[41] Alliot C, Barrios M, Taib J, Brunel M (2001). Parvovirus B19 infection in an HIV-infected patient with febrile pancytopenia and acute hepatitis. *European Journal of Clinical Microbiology and Infectious Diseases*.

[42] Karetnyi YV, Beck PR, Markin RS, Langnas AN, Naides SJ (1999). Human parvovirus B19 infection in acute fulminant liver failure. *Archives of Virology*.

[43] Drago F, Semino M, Rampini P, Rebora A (1999). Parvovirus B19 infection associated with acute hepatitis and a purpuric exanthem. *British Journal of Dermatology*.

[44] Sokal EM, Melchior M, Cornu C (1998). Acute parvovirus B19 infection associated with fulminant hepatitis of favourable prognosis in young children. *The Lancet*.

[45] Hillingsø JG, Jensen IP, Tom-Petersen L (1998). Parvovirus B19 as causative agent of acute hepatitis in adults. *Ugeskrift for Laeger*.

[46] Hillingsø JG, Jensen IP, Tom-Petersen L (1998). Parvovirus B19 and acute hepatitis in adults. *The Lancet*.

[47] Longo G, Luppi M, Bertesi M, Ferrara L, Torelli G, Emilia G (1998). Still’s disease, severe thrombocytopenia, and acute hepatitis associated with acute parvovirus B19 infection. *Clinical Infectious Diseases*.

[48] Pardi DS, Romero Y, Mertz LE, Douglas DD (1998). Hepatitis-associated aplastic anemia and acute parvovirus B19 infection: a report of two cases and a review of the literature. *The American Journal of Gastroenterology*.

[49] Weinberg JM, Wolfe JT, Frattali AL, Werth VP, Naides SJ, Spiers EM (1996). Parvovirus B19 infection associated with acute hepatitis, arthralgias, and rash. *Journal of Clinical Rheumatology*.

[50] Naides SJ, Karetnyi YV, Cooling LLW (1996). Human parvovirus B19 infection and hepatitis. *The Lancet*.

[51] Yoto Y, Kudoh T, Haseyama K, Suzuki N, Chiba S (1996). Human parvovirus B19 infection associated with acute hepatitis. *The Lancet*.

[52] Langnas AN, Markin RS, Cattral MS, Naides SJ (1995). Parvovirus B19 as a possible causative agent of fulminant liver failure and associated aplastic anemia. *Hepatology*.

[53] Pouchot J, Ouakil H, Debin ML, Vinceneux P (1993). Adult Still’s disease associated with acute human parvovirus B19 infection. *The Lancet*.

[54] Toan NL, Song LH, Kremsner PG (2006). Co-infection of human parvovirus B19 in Vietnamese patients with hepatitis B virus infection. *Journal of Hepatology*.

[55] Hsu T-C, Chen T-Y, Lin M-C, Tzang B-S, Tsay GJ (2005). Human parvovirus B19 infection in patients with chronic hepatitis B or hepatitis C infection. *Journal of Gastroenterology and Hepatology*.

[56] Dwivedi M, Manocha H, Tiwari S, Tripathi G, Dhole TN (2009). Coinfection of parvovirus b19 with other hepatitis viruses leading to fulminant hepatitis of unfavorable outcome in children. *The Pediatric Infectious Disease Journal*.

[57] Osugi Y, Yagasaki H, Sako M (2007). Antithymocyte globulin and cyclosporine for treatment of 44 children with hepatitis associated aplastic anemia. *Haematologica*.

[58] Rauff B, Idrees M, Shah SAR (2011). Hepatitis associated aplastic anemia: a review. *Virology Journal*.

[59] Andreesen R, Brugger W, Thomssen C, Rehm A, Speck B, Lohr GW (1989). Defective monocyte-to-macrophage maturation in patients with aplastic anemia. *Blood*.

[60] Muta T, Tanaka Y, Takeshita E (2008). Recurrence of hepatitis-associated aplastic anemia after a 10-year Interval. *Internal Medicine*.

[61] Zaki MES, Hassan SA, Seleim T, Lateef RA (2006). Parvovirus B19 infection in children with a variety of hematological disorders. *Hematology*.

[62] Serke S, Schwarz TF, Baurmann H (1991). Productive infection of in vitro generated haemopoietic progenitor cells from normal human adult peripheral blood with parvovirus B19: studies by morphology, immunocytochemistry, flow-cytometry and DNA-hybridization. *British Journal of Haematology*.

[63] Kudoh T, Yoto Y, Suzuki N (1994). Human parvovirus B19-induced aplastic crisis in iron deficiency anemia. *Acta Paediatrica Japonica*.

[64] Janka G (2009). Hemophagocytic lymphohistiocytosis: when the immune system runs amok. *Klinische Padiatrie*.

[65] Hoang MP, Dawson DB, Rogers ZR, Scheuermann RH, Rogers BB (1998). Polymerase chain reaction amplification of archival material for Epstein-Barr virus, cytomegalovirus, human herpesvirus 6, and parvovirus B19 in children with bone marrow hemophagocytosis. *Human Pathology*.

[66] Shirono K, Tsuda H (1995). Parvovirus B19-associated haemophagocytic syndrome in healthy adults. *British Journal of Haematology*.

[67] Su I-J, Wang C-H, Cheng A-L, Chen R-L (1995). Hemophagocytic syndrome in Epstein-Barr virus-associated T-lymphoproliferative disorders: disease spectrum, pathogenesis, and management. *Leukemia and Lymphoma*.

[68] Mustafa MM, McClain KL (1996). Diverse hematologic effects of parvovirus B19 infection. *Pediatric Clinics of North America*.

[69] Smith-Whitley K, Zhao H, Hodinka RL (2004). Epidemiology of human parvovirus B19 in children with sickle cell disease. *Blood*.

[70] Pickering LK, Baker CJ, Kimberlin DW, Long SS, The American Academy of Pediatrics Committee on Infectious Diseases (2009). Parvovirus B19. *Red Book: Report of the Committee on Infectious Diseases*.

[71] Fairley CK, Smoleniec JS, Caul OE, Miller E (1995). Observational study of effect of intrauterine transfusions on outcome of fetal hydrops after parvovirus B19 infection. *The Lancet*.

[72] Söderlund-Venermo M, Hokynar K, Nieminen J, Rautakorpi H, Hedman K (2002). Persistence of human parvovirus B19 in human tissues. *Pathologie Biologie*.

[73] Mandell GL, Bennet JE, Dolin R (2005). *Mandell, Douglas and Bennett's Principals and Practice of Infectious Diseases*.

[74] Morita E, Nakashima A, Asao H, Sato H, Sugamura K (2003). Human parvovirus B19 nonstructural protein (NS1) induces cell cycle arrest at G1 phase. *Journal of Virology*.

[75] Cooling LLW, Koerner TAW, Naides SJ (1995). Multiple glycosphingolipids determine the tissue tropism of parvovirus B19. *Journal of Infectious Diseases*.

[76] Poole BD, Karetnyi YV, Naides SJ (2004). Parvovirus B19-induced apoptosis of hepatocytes. *Journal of Virology*.

[77] Young NS, Maciejewski J (1997). The pathophysiology of acquired aplastic anemia. *The New England Journal of Medicine*.

[78] Doney K, Leisenring W, Storb R, Appelbaum FR (1997). Primary treatment of acquired aplastic anemia: outcomes with bone marrow transplantation and immunosuppressive therapy. *Annals of Internal Medicine*.

